# Next Generation Sequencing and Genetic Alterations in Squamous Cell Lung Carcinoma: Where Are We Today?

**DOI:** 10.3389/fonc.2019.00166

**Published:** 2019-03-19

**Authors:** Alex Friedlaender, Giuseppe Banna, Umberto Malapelle, Pasquale Pisapia, Alfredo Addeo

**Affiliations:** ^1^Oncology Department, Geneva University Hospital, Geneva, Switzerland; ^2^Oncology Department, Ospedale Cannizzaro, Catania, Italy; ^3^Department of Public Health, University of Naples Federico II, Naples, Italy

**Keywords:** NGS—next generation sequencing, squamous cell lung cancer (SQCLC), targeted therapy (TT), FGFR1 amplification, PI3 K, non-small cell lung cancer, *MET*, genetic alterations

## Abstract

Lung cancer is the leading cause of cancer-related mortality and will affect ~6% of the population. It is divided into two broad categories, small cell lung cancer and non-small cell lung cancer (NSCLC), the latter representing 85% of all lung cancers. It mainly comprises adenocarcinoma (65%) and squamous cell carcinoma (30%) histologies. In recent years, there have been two major therapeutic advances in NSCLC. The first, immunotherapy, has greatly improved the prognosis of adenocarcinomas and squamous cell carcinomas. The second, the treatment of targetable driver mutations, has so far only benefited adenocarcinomas. Squamous cell carcinoma carries a high rate of mutations and is found mostly among smokers. This raises two important problems: identifying driver mutations and finding those of clinical relevance. Large-scale genomic analyses such as The Cancer Genome Atlas have allowed for the identification of frequent gene alterations, although their role and potential for targeted therapy remain unknown. The emergence of next generation sequencing has changed the landscape of precision medicine, in particular in lung cancer. In this review, we discuss the landscape of genetic alterations found in squamous cell lung cancer, the results of current targeted therapy trials, the difficulties in identifying and treating these alterations and how to integrate modern tools in clinical practice.

## Introduction

Lung cancer is the leading cause of cancer-related mortality and affects ~6% of the population. It is divided into two broad categories, small cell lung cancer and non-small cell lung cancer (NSCLC). The latter represents 85% of all lung cancers. NSCLC comprises mainly adenocarcinoma (ADC) (60%) and squamous cell carcinoma (SqCC) (35%) histological subtypes (1), each with separate mutational and genomic profiles.

In the era of personalized medicine, next generation sequencing (NGS) plays a key role in assessing the molecular status and administering the best treatment choice for each patient. NGS allows a comprehensive multi-gene analysis and facilitates the identification of recurrent alterations for targeted therapy. Essentially, large numbers of DNA fragments are bound to arrays and sequenced in parallel. The bio-informatic analysis allows precise comparisons to reference genomes. Before NGS, genomic analyses were limited to specific loci known to be associated with each cancer subtype. Single-gene sequencing like the Sanger technique is limited to DNA insertions, deletions, and substitutions, while NGS has the potential to further detect chromosomal rearrangements, oncogenic fusion events, translocations and copy number alterations (2, 3). It is noteworthy that NGS is more cost-effective than sequential single-gene tests for the main alterations in non-squamous NSCLC (4). Furthermore, it has a higher sensitivity and specificity, with a potential impact on therapeutic sequences (2, 3).

There has been significant improvement in ADC treatment choice in recent years thanks to the identification of targetable mutations that lead to oncogenesis, known as driver mutations. These are more frequent among non-smokers and younger patients and confer a clear survival benefit when treated with targeted therapies (5–7). Genetic alterations and their products can be identified through various techniques including immunohistochemistry (IHC), fluorescence *in situ* hybridization (FISH) and NGS. For ADCs, the most common therapeutic targets are *EGFR* and *BRAF* mutations, *ALK* and *ROS1* rearrangements, with others such as *MET, RET, NTRK, HER2* showing various degrees of response in clinical trials (8–10). In a comprehensive NGS-based genomic study of 10,472 advanced lung ADC patients, over 40% of cancers had druggable alterations (11). Today, the number of these targets analyzable by NGS is ever-growing. SqCC represents 30% of NSCLC worldwide (12). Less evidence is currently available on genetic targets in SqCC.

This review will focus on genomic profiling and targeted treatments in SqCC.

## SqCC and the Emerging Evidence and Hurdles of NGS

SqCC offers a very different picture. It is a disease found mostly among smokers and carries a high rate of mutations (13). This raises two important problems: identifying driver mutations and finding those of clinical relevance. As mentioned, the emergence of NGS has changed the landscape of precision medicine, in particular in lung cancer (14–16). Large-scale genomic analyses such as The Cancer Genome Atlas have collected data on many tumor types and allowed the identification of frequent gene alterations, although their role and potential for targeted therapy remain unknown (17). As shown by Schwaederle et al. squamous histotypes arising in different anatomical sites feature the existence of genomic patterns for the so called “squamousness.” Through an NGS approach, the authors identified frequent mutations in *TP53* (64.5% of analyzed patients), *PIK3CA* (28.5%), *CDKN2A* (24.4%), *SOX2* (17.7%), and *CCND1* (15.8%) (18). Given its incidence, the amplification of the transcription factor SOX2 is particularly interesting. Chromosome 3q amplification, represents the most common genomic aberration that plays a role in the evolution of pre-invasive SqCC. SOX2 is a “lineage-survival oncogene” and its activity promotes the differentiation into and proliferation of squamous cells instead of a loss of cellular differentiation. Interestingly, the transcription factor SOX2 is the predominant downstream target of the EGFR signaling pathway and plays a major role in self-renewal, growth, and expansion of cell populations. In light of the complex actions of SOX2 in regulating normal and tumor cell development, the elucidation of SOX2-dependent pathways may identify new therapeutic vulnerabilities in lung cancer. However, because of their lack of small molecule binding pockets, transcription factors are currently an example of “undruggable targets.”

Thus, unlike in ADC, there are currently very few actionable or druggable mutations in SqCC, which remains a challenging disease to treat. Until recently, the backbone of SqCC therapy was chemotherapy but fortunately, there has been significant progress. Current first-line treatment of metastatic SqCC is a platinum-based chemotherapy doublet, immunotherapy or a combination thereof, while second-line treatment is immunotherapy, single-agent chemotherapy with or without anti-angiogenic, or anti-EGFR tyrosine kinase inhibitors, the latter two with a marginal clinical impact (19, 20). Thereafter, supportive care is currently the best option. As first-line treatment evolves, it leaves us with an ever-shrinking arsenal for subsequent therapies and highlights the desperate need for progress. Much work is underway to elucidate potential treatments, but as we will now explain, this is anything but an easy feat.

A recent publication performed an interesting secondary analysis among advanced SqCC patients on second-line therapy in the LUX-Lung 8 trial, attempting to use the NGS to identify whether patients with *ERBB* gene alterations derive increased benefit from the anti-*EGFR* tyrosine kinase inhibitor afatinib compared to erlotinib (21). This study had a number of confounding factors including the fact that it analyzed only 31% of the intention-to-treat population, the majority enriched with a prolonged progression-free survival (PFS), representing a selection bias. Though this analysis was planned at the start of the trial, NGS was not feasible in a large proportion of specimens, highlighting the importance of collecting adequate tissue samples if genomic analyses are warranted. This issue may also be shared with tumor mutation burden (TMB), should this biomarker become standard practice. The trial revealed 21.6% *ERBB* family mutations, with an intriguing yet non-statistically significant overall survival (OS) difference in favor of the afatinib group. Given the small number and the above-mentioned selection bias, it seems imprudent to assume the difference is relevant.

Furthermore, this study highlights a recurrent difficulty in the interpretation of NGS analyses. Due to the small number of patients, the authors had to group all the *ERBB* mutations together, but in doing so, have likely mixed passenger mutations with real driver ones, diluting the impact of potential targets of clinical relevance.

This paper highlights the need to identify driver mutations in SqCC. *ERBB* mutations in NSCLC are well-known but results have been rather disappointing, raising the possibility that they may not represent an actionable target (22).

In a study by Lindquist et al. NGS analysis revealed that 13% of SqCC harbor at least one potentially actionable alteration (23).

## What About Other Frequent Alterations Detected in NGS in SqCC?

While ERBB mutations have yet to prove their clinical relevance in SqCC, other targets appear promising. While the most frequent mutation in SqCC is the unactionable TP53, other common potentially targetable alterations include the fibroblast growth factor receptor 1 (FGFR1) amplification and phosphatidylinositol 3-kinase (PI3K) abnormalities (Table 1) (13).

**Table 1:** Current potentially actionable genetic alterations in SqCC detected by NGS.

**Gene**	**Clinical findings**	**Note**
ERBB	ERBB family mutations in 21.6% of SqCC, non-statistically significant overall survival difference in favor of afatinib vs. erlotinib (LUX-Lung 8 trial)	Difficulties in discerning mixed passenger mutations from real driver ones
FGFR1	Amplification in 20% of SqCC, no correlation between amplification and increased protein expression, better prognosis independent of treatment, no survival benefit in unselected advanced SqCC patients (LUME-Lung 1 trial) in adding the FGFR inhibitor nintedanib to docetaxel in second-line therapy	Role pending in ongoing large international studies
PI3K	Missense mutations and amplifications, in ~20% of advanced SqCC, frequently associated with loss of PTEN, worse prognosis, buparlisib showed poor disease responses with only 20% PFS at 12 weeks among NSCLC patients with *PIK3CA* activating mutations	Seems not to be a driver mutation

*FGFR1, fibroblast growth factor receptor; NGS, next-generation sequencing (NGS); PI3K, phosphatidylinositol 3-kinase; SqCC, squamous cell carcinoma*.

The TP53 gene plays an important role in the tumorigenesis of epithelial lung cells. Genetic abnormalities of TP53 in lung cancer are associated with increased cellular resistance to therapy (24). It is likely the most extensively investigated prognostic marker in NSCLC, with a modest negative prognostic role. Unfortunately, no targeted treatments have been proven effective for TP53.

FGFR1 amplification can be identified in approximately 20% of SqCC (25), and is rare in ADC. It is linked to smoking but independent of age and pathological features. Biologically, it is an interesting target. It is a tyrosine kinase whose activation downregulates the phosphatidylinositol 3-kinase/v-Akt (PI3K/AKT) reticular activating system/mitogen-activated protein kinase (RAS/MAPK) pathways, hindering cell growth and angiogenesis. Epidermal growth factor receptor (EGFR) and platelet-derived growth factor receptor (PDGFR) are the best-known receptors in the RAS/MAPK pathway. Thus, as with EGFR, several studies are ongoing to better define whether PDGFR is a therapeutic target.

Preclinical data is promising, with good responses in xenografts of FGFR1 amplified cell lines to FGFR inhibitors (26). However, in clinical trials, efficacy has been limited and heterogeneous. Unlike in the previous example, the target is consistent, being an FGFR1 amplification and not a general family of alterations. In spite of this, there is not a uniform correlation between amplification and increased protein expression (27). In other words, genotype does not equate phenotype, and this could explain the diverging clinical impact of inhibitory drugs. In unselected advanced SqCC patients in the LUME-Lung 1 trial, adding the FGFR inhibitor nintedanib to docetaxel in second-line therapy had no survival benefit (28). Large international studies are underway to conclusively answer the role of FGFR1 amplification and its potential as a target in SqCC (29). Finally, it is interesting to note that NGS analysis has also allowed us to identify that FGFR1 amplification seems to be linked with better prognosis, independent of treatment (25).

PI3K alterations, mainly missense mutations and amplifications, are also frequent in SqCC, found in ~20% of advanced diseases (25). Alterations of the PI3K/AKT/mTOR pathway can occur at many levels resulting in PI3K activation and malignant transformation. They are also frequently associated with loss of tumor suppressor *PTEN*. This well-known cellular signaling pathway regulates cell growth, survival and metabolism (30). While much research exists on targeted treatments, there seems to be little correlation between molecular alterations and response rate. Preclinical data reveal significant *in vivo* pro-apoptotic and anti-proliferative effects of *PIK3CA* inhibitors (31). In breast cancer, the PI3K pathway represents a mechanism of endocrine therapy resistance (32). In advanced endocrine therapy-resistant breast cancer, the addition of buparlisib prolongs PFS, though no correlation with *PIK3CA* mutation was demonstrated (33). Similarly, glioblastomas, of which ~50% have PI3K pathway alterations, can show stable disease in 20% of cases when treated with targeted therapy, yet PI3K alterations are not predictive biomarkers of response (34). Finally, in NSCLC, the impact of PI3K inhibitors among patients with PI3K alterations seems negligible. The BASALT-1 phase II trial assessed the efficacy of buparlisib among NSCLC patients with *PIK3CA* activating mutations. Both ADCs and SqCC showed poor disease responses with only 20% PFS at 12 weeks (35). It is interesting to note that while the question of whether this is a useful target for precision medicine remains unanswered, this alteration has a major prognostic impact, with a median OS of less than half that of patients without PI3K aberrations. These patients also more frequently develop multiple-organ progression and brain metastases, though the latter is usually accompanied by the loss of the tumor suppressor PTEN protein (25).

Finally, when faced with these poor results and the lack of a useful biomarker, some questions beckon: is the PI3K pathway simply one of many simultaneous driver mutations? Are we treating the right target?

These examples only serve to illustrate the complexity of identifying and properly investigating potential targets in SqCC. The Lung-MAP study is a collaborative international study comprising multiple phase II studies, each for a specific biomarker identified in SqCC. All participating patients who progress after first-line therapy have NGS analysis of their tumor. It is an umbrella study that aims to evaluate multiple targeted therapeutic strategies in a single type of cancer. Lung-MAP does not use adaptive randomization to evaluate drug-biomarker combinations and goes beyond phase II development. It has been designed to provide a path for FDA approval of active agents identified in the initial phase II study. That is, a drug that is found to be effective in phase II will move directly into the phase III registration setting, incorporating the patients from phase II. This will reduce time, resources, and patient numbers needed to accomplish the ultimate goal of bringing novel agents to the clinic. Lung-MAP might also address other unmet needs, including applications of broad-based genomic screening in clinical trial settings and shortened turnaround times to allow effective use of molecular testing in treatment-selection for rapidly progressing patients. This master protocol mechanism might improve access to genomic screening for SqCC patients, improve the identification of genomic biomarkers for clinical trial entry, and accelerate drug–biomarker testing. The primary study endpoint is disease response rates in the second-line SqCC and it will lead to larger phase III studies in case of compelling results (36).

The *MET* signaling pathway is often dysregulated in solid malignancies, including lung cancer, as a result of several mechanisms such as autocrine/paracrine stimulation, *MET* overexpression, genomic amplification, translocations, point mutations and alternative splicing (37). *MET* overexpression has been reported in 29% of the SqCC patients and is associated with a poor prognosis (38).

In a cohort of 262 lung cancer patients that included predominantly NSCLC and only 2 SCLC, all instances of *MET* activation occurred in adenocarcinomas (39). The prevalence of *MET* gene amplification and splice mutations were 1.4 and 3.3%, respectively.

A humanized *MET* monoclonal antibody, onartuzumab, specifically designed to block HGF-induced *MET* dimerization and activation of the intracellular kinase domain was tested in a phase II study in recurrent NSCLC. *MET*-positive patients treated with erlotinib plus onartuzumab showed improvement in both PFS [harzad ratio (HR) 0.53; *P* = 0.04] and OS (HR 0.37; *P* = 0.002) (40). Despite these encouraging results, the addition of onartuzumab to erlotinib did not improve median OS (6.8 vs. 9.1 months), PFS (2.7 vs. 2.6 months), or overall response rate (8.4% vs. 9.6%) in previously treated stage IIIb or IV NSCLC in the phase 3 trial (41). The most frequent adverse events that were higher in the combination arm were peripheral edema, hypoalbuminemia, back pain, dyspnea, nausea, acneiform dermatitis, and rash. The efficacy of onartuzumab in gastric cancer was also disappointing. The reason multiple clinical trials targeting *MET* have failed seems to be drug design and/or patient selection. Onartuzumab was designed to block HGF-MET interaction by targeting the beta subunit of extracellular Sema domain of *MET*, required for HGF binding, but likely unnecessary for *MET* dimerization. *MET*-amplified tumor cells normally exhibit ligand-independent, constitutive *MET* activation (42). Thus, in cancers driven by *MET* amplification/overexpression or activating mutations, *MET* activation and downstream signaling is unlikely to be fully blocked by drugs solely targeting HGF-MET binding. In addition, most recent late phase trials recruited *MET*-high patients. *MET*-high lung tumors, corresponding to IHC 2+ or 3+, likely carry *MET* amplifications and are probably HGF-independent. This could explain why they did not respond to therapies that only target HGF-MET binding.

Hammerman et al. identified the *DDR2* gene mutation in 4% of SqCC, with a sensitivity to dasatinib (43).

## Targeted Treatments in SqCC

In addition to those described above, other studies showed the possibility of administering targeted therapy in SqCC patients. It is important to note that the benefits described are quite limited. In the FLEX trial, a survival improvement was seen in the *EGFR*-expressing advanced SqCC subgroup after the administration of cetuximab plus chemotherapy (44). In the SQUIRE trial, the authors showed that the addition of necitumumab to gemcitabine and cisplatin could improve OS in patients with advanced SqCC with high *EGFR* expression (45). The role of immunotherapy in SqCC has been well-documented. Among previously treated advanced SqCC patients in CheckMate 017, there was an increase in response rate, PFS and OS with nivolumab with respect to docetaxel, regardless of PD-L1 expression (46). In KEYNOTE-010, pembrolizumab was tested in NSCLC patients, regardless of histology. Subgroup analysis of SqCC patients revealed a non-significant benefit with respect to docetaxel (47). In the POPLAR study, atezolizumab showed a significant OS improvement compared to docetaxel in 287 pre-treated squamous or non-squamous NSCLC patients (48).

Recently several studies have changed the first-line treatment landscape. The combination of chemotherapy plus check-point inhibitors (ICI) is superior in term of OS and PFS to chemotherapy alone in metastatic SqCC (49–52). It has also been demonstrated that immunotherapy is not effective as a first line in NSCLC patients harboring driver mutations (53, 54). Thus, correctly identifying possible driver mutations in SqCC might lead to improved efficacy of ICI by omitting this treatment in a subgroup of patients who should not be exposed first-line ICI.

## Conclusion

In the era of personalized medicine, SqCC is lagging far behind ADC but change is coming. Today, the initially most promising targets appear disappointing in SqCC. However, we must be prudent in the manner in which therapeutic targets are chosen, as to avoid overlooking real driver mutations by grouping many alterations together. Similarly, the choice of drugs and potential combinations will play an important role. Promising trials like Lung-MAP will allow for a better understanding of the clinical relevance of suspected driver mutations. Finally, while NGS technology offers a wide array of possibilities, we believe it is through trials that this approach will evolve and not through sporadic NGS analysis and off-label personalized medicine to every patient, without any evidence of therapeutic utility. It is important to remember our Hippocratic Oath: *primum non nocere*, first, do no harm, and targeted therapies are not without risk (or cost).

## Author Contributions

All authors listed have made a substantial, direct and intellectual contribution to the work, and approved it for publication.

### Conflict of Interest Statement

The authors declare that the research was conducted in the absence of any commercial or financial relationships that could be construed as a potential conflict of interest.

## References

[1] Howlader N Noone AM Krapcho M Miller D Bishop K Kosary CL SEER Cancer Statistics Review, 1975-2014. National Cancer Institute. Bethesda, MD (2017). Available online at: https://seer.cancer.gov/csr/1975_2014/

[2] Gagan J Van Allen EM. Next-generation sequencing to guide cancer therapy. Genome Med. (2015) 7:80. 10.1186/s13073-015-0203-x 26221189 PMC4517547

[3] Lu YQ Lu KH. Advancements in next-generation sequencing for diagnosis and treatment of non-small-cell lung cancer. Chronic Dis Transl Med. (2017) 3:1–7. 10.1016/j.cdtm.2017.02.009 29063051 PMC5627693

[4] Pennell NA Mutebi A Zhou Z-Y Ricculli ML Tang W Wang H Economic impact of next generation sequencing vs. sequential single-gene testing modalities to detect genomic alterations in metastatic non-small cell lung cancer using a decision analytic model. J Clin Oncol. (2018) 36(Suppl. 15):9031 10.1200/JCO.2018.36.15_suppl.9031 35100695

[5] Mok TS Wu YL Thongprasert S Yang CH Chu DT Saijo N. Gefitinib or carboplatin-paclitaxel in pulmonary adenocarcinoma. N Engl J Med. (2009) 361:947–57. 10.1056/NEJMoa0810699 19692680

[6] Rosell R Carcereny E Gervais R Vergnenegre A Massuti B Felip E. Erlotinib versus standard chemotherapy as first-line treatment for European patients with advanced EGFR mutation-positive non-small-cell lung cancer (EURTAC): a multicentre, open-label, randomised phase 3 trial. Lancet Oncol. (2012) 13:239–46. 10.1016/S1470-2045(11)70393-X 22285168

[7] Yang JC Wu YL Schuler M Sebastian M Popat S Yamamoto N. Afatinib versus cisplatin-based chemotherapy for EGFR mutation-positive lung adenocarcinoma (LUX-Lung 3 and LUX-Lung 6): analysis of overall survival data from two randomised, phase 3 trials. Lancet Oncol. (2015) 16:141–51. 10.1016/S1470-2045(14)71173-8 25589191

[8] Landi L Tiseo M Chiari R Ricciardi S Rossi E Galetta D. Activity of the EGFR-HER2 dual inhibitor afatinib in EGFR-mutant lung cancer patients with acquired resistance to reversible EGFR tyrosine kinase inhibitors. Clin Lung Cancer. (2014) 15:411–17 e414. 10.1016/j.cllc.2014.07.002 25242668

[9] Addeo A Tabbo F Robinson T Buffoni L Novello S. Precision medicine in ALK rearranged NSCLC: a rapidly evolving scenario. Crit Rev Oncol Hematol. (2018) 122:150–6. 10.1016/j.critrevonc.2017.12.015 29458783

[10] Pilotto S Rossi A Vavala T Follador A Tiseo M Galetta D. Outcomes of first-generation EGFR-TKIs against non-small-cell lung cancer harboring uncommon EGFR mutations: a *post hoc* analysis of the BE-POSITIVE Study. Clin Lung Cancer. (2018) 19:93–104. 10.1016/j.cllc.2017.05.016 28645631

[11] Gupta A Connelly C Frampton G Chmielecki J Ali S Suh J P2.03b-068 the druggable mutation landscape of lung adenocarcinoma: topic: biomarkers. J Thoracic Oncol. (2017) 12:S977 10.1016/j.jtho.2016.11.1349

[12] Perez-Moreno P Brambilla E Thomas R Soria JC. Squamous cell carcinoma of the lung: molecular subtypes and therapeutic opportunities. Clin Cancer Res. (2012) 18:2443–51. 10.1158/1078-0432.CCR-11-2370 22407829

[13] Okamoto T Takada K Sato S Toyokawa G Tagawa T Shoji F. Clinical and genetic implications of mutation burden in squamous cell carcinoma of the lung. Ann Surg Oncol. (2018) 25:1564–71. 10.1245/s10434-018-6401-1 29500766

[14] Malapelle U Mayo de-Las-Casas C Rocco D Garzon M Pisapia P Jordana-Ariza N. Development of a gene panel for next-generation sequencing of clinically relevant mutations in cell-free DNA from cancer patients. Br J Cancer. (2017) 116:802–10. 10.1038/bjc.2017.8 28170370 PMC5355934

[15] Pisapia P Pepe F Smeraglio R Russo M Rocco D Sgariglia R. Cell free DNA analysis by SiRe((R)) next generation sequencing panel in non small cell lung cancer patients: focus on basal setting. J Thorac Dis. (2017) 9(Suppl. 13):S1383–90. 10.21037/jtd.2017.06.97 29184677 PMC5676109

[16] Pepe F De Luca C Smeraglio R Pisapia P Sgariglia R Nacchio M. Performance analysis of SiRe next-generation sequencing panel in diagnostic setting: focus on NSCLC routine samples. J Clin Pathol. (2019) 72:38–45. 10.1136/jclinpath-2018-205386 30279174

[17] Cancer Genome Atlas Research Network Comprehensive genomic characterization of squamous cell lung cancers. Nature. (2012) 489:519–25. 10.1038/nature11404 22960745 PMC3466113

[18] Schwaederle M Elkin SK Tomson BN Carter JL Kurzrock R. Squamousness: next-generation sequencing reveals shared molecular features across squamous tumor types. Cell Cycle. (2015) 14:2355–61. 10.1080/15384101.2015.1053669 26030731 PMC4613537

[19] Postmus PE Kerr KM Oudkerk M Senan S Waller DA Vansteenkiste J. Early and locally advanced non-small-cell lung cancer (NSCLC): ESMO Clinical Practice Guidelines for diagnosis, treatment and follow-up. Ann Oncol. (2017) 28(Suppl. 4):iv1–21. 10.1093/annonc/mdx222 28881918

[20] Banna GL Passiglia F Colonese F Canova S Menis J Addeo A. Immune-checkpoint inhibitors in non-small cell lung cancer: a tool to improve patients' selection. Crit Rev Oncol Hematol. (2018) 129:27–39. 10.1016/j.critrevonc.2018.06.016 30097235

[21] Goss GD Felip E Cobo M Lu S Syrigos K Lee KH. Association of ERBB mutations with clinical outcomes of afatinib- or erlotinib-treated patients with lung squamous cell carcinoma: secondary analysis of the LUX-lung 8 randomized clinical trial. JAMA Oncol. (2018) 4:1189–97. 10.1001/jamaoncol.2018.0775 29902295 PMC6143014

[22] Kris MG Camidge DR Giaccone G Hida T Li BT O'Connell J Targeting HER2 aberrations as actionable drivers in lung cancers: phase II trial of the pan-HER tyrosine kinase inhibitor dacomitinib in patients with HER2-mutant or amplified tumors. Ann Oncol. (2015) 26:1421–7. 10.1093/annonc/mdv186 25899785 PMC5006511

[23] Lindquist KE Karlsson A Leveen P Brunnstrom H Reutersward C Holm K. Clinical framework for next generation sequencing based analysis of treatment predictive mutations and multiplexed gene fusion detection in non-small cell lung cancer. Oncotarget. (2017) 8:34796–810. 10.18632/oncotarget.16276 28415793 PMC5471012

[24] Baumann M Zips D Appold S. Radiotherapy of lung cancer: technology meets biology meets multidisciplinarity. Radiother Oncol. (2009) 91:279–81. 10.1016/j.radonc.2009.05.001 19464524

[25] Paik PK Shen R Won H Rekhtman N Wang L Sima CS. Next-generation sequencing of stage IV squamous cell lung cancers reveals an association of PI3K aberrations and evidence of clonal heterogeneity in patients with brain metastases. Cancer Discov. (2015) 5:610–21. 10.1158/2159-8290.CD-14-1129 25929848 PMC4643059

[26] Zhang J Zhang L Su X Li M Xie L Malchers F. Translating the therapeutic potential of AZD4547 in FGFR1-amplified non-small cell lung cancer through the use of patient-derived tumor xenograft models. Clin Cancer Res. (2012) 18:6658–67. 10.1158/1078-0432.CCR-12-2694 23082000

[27] Wynes MW Hinz TK Gao D Martini M Marek LA Ware KE. FGFR1 mRNA and protein expression, not gene copy number, predict FGFR TKI sensitivity across all lung cancer histologies. Clin Cancer Res. (2014) 20:3299–309. 10.1158/1078-0432.CCR-13-3060 24771645 PMC4062100

[28] Reck M Kaiser R Mellemgaard A Douillard JY Orlov S Krzakowski M. Docetaxel plus nintedanib versus docetaxel plus placebo in patients with previously treated non-small-cell lung cancer (LUME-Lung 1): a phase 3, double-blind, randomised controlled trial. Lancet Oncol. (2014) 15:143–55. 10.1016/S1470-2045(13)70586-2 24411639

[29] Hashemi-Sadraei N Hanna N. Targeting FGFR in squamous cell carcinoma of the lung. Target Oncol. (2017) 12:741–55. 10.1007/s11523-017-0513-6 28685321

[30] Engelman JA. Targeting PI3K signalling in cancer: opportunities, challenges and limitations. Nat Rev Cancer. (2009) 9:550–62. 10.1038/nrc2664 19629070

[31] Maira SM Pecchi S Huang A Burger M Knapp M Sterker D. Identification and characterization of NVP-BKM120, an orally available pan-class I PI3-kinase inhibitor. Mol Cancer Ther. (2012) 11:317–28. 10.1158/1535-7163.MCT-11-0474 22188813

[32] Ciruelos Gil EM Targeting the PI3K/AKT/mTOR pathway in estrogen receptor-positive breast cancer. Cancer Treat Rev. (2014) 40:862–71. 10.1016/j.ctrv.2014.03.004 24774538

[33] Baselga J Im SA Iwata H Cortes J De Laurentiis M Jiang Z. Buparlisib plus fulvestrant versus placebo plus fulvestrant in postmenopausal, hormone receptor-positive, HER2-negative, advanced breast cancer (BELLE-2): a randomised, double-blind, placebo-controlled, phase 3 trial. Lancet Oncol. (2017) 18:904–16. 10.1016/S1470-2045(17)30376-5 28576675 PMC5549667

[34] Pitz MW Eisenhauer EA MacNeil MV Thiessen B Easaw JC Macdonald DR. Phase II study of PX-866 in recurrent glioblastoma. Neuro Oncol. (2015) 17:1270–4. 10.1093/neuonc/nou365 25605819 PMC4588751

[35] Vansteenkiste JF Canon JL De Braud F Grossi F De Pas T Gray JE. Safety and efficacy of Buparlisib (BKM120) in patients with PI3K pathway-activated non-small cell lung cancer: results from the phase II BASALT-1 Study. J Thorac Oncol. (2015) 10:1319–27. 10.1097/JTO.0000000000000607 26098748 PMC4646607

[36] Herbst RS Gandara DR Hirsch FR Redman MW LeBlanc M Mack PC. Lung Master Protocol (Lung-MAP)-a biomarker-driven protocol for accelerating development of therapies for squamous cell lung cancer: SWOG S1400. Clin Cancer Res. (2015) 21:1514–24. 10.1158/1078-0432.CCR-13-3473 25680375 PMC4654466

[37] Gherardi E Birchmeier W Birchmeier C Vande Woude G. Targeting *MET* in cancer: rationale and progress. Nat Rev Cancer. (2012) 12:89–103. 10.1038/nrc3205 22270953

[38] Dziadziuszko R Wynes MW Singh S Asuncion BR Ranger-Moore J Konopa K. Correlation between *MET* gene copy number by silver *in situ* hybridization and protein expression by immunohistochemistry in non-small cell lung cancer. J Thorac Oncol. (2012) 7:340–7. 10.1097/JTO.0b013e318240ca0d 22237262 PMC3358920

[39] Onozato R Kosaka T Kuwano H Sekido Y Yatabe Y Mitsudomi T. Activation of *MET* by gene amplification or by splice mutations deleting the juxtamembrane domain in primary resected lung cancers. J Thorac Oncol. (2009) 4:5–11. 10.1097/JTO.0b013e3181913e0e 19096300

[40] Spigel DR Ervin TJ Ramlau RA Daniel DB Goldschmidt JH Jr Blumenschein GR Jr. Randomized phase II trial of Onartuzumab in combination with erlotinib in patients with advanced non-small-cell lung cancer. J Clin Oncol. (2013) 31:4105–14. 10.1200/JCO.2012.47.4189 24101053 PMC4878106

[41] Spigel DR Edelman MJ O'Byrne K Paz-Ares L Shames DS Yu W Onartuzumab plus erlotinib versus erlotinib in previously treated stage IIIb or IV NSCLC: results from the pivotal phase III randomized, multicenter, placebo-controlled *MET*Lung (OAM4971g) global trial. J Clin Oncol. (2014) 32(Suppl. 15):8000 10.1200/jco.2014.32.15_suppl.8000

[42] Kong-Beltran M Stamos J Wickramasinghe D. The Sema domain of *MET* is necessary for receptor dimerization and activation. Cancer Cell. (2004) 6:75–84. 10.1016/j.ccr.2004.06.013 15261143

[43] Hammerman PS Sos ML Ramos AH Xu C Dutt A Zhou W. Mutations in the DDR2 kinase gene identify a novel therapeutic target in squamous cell lung cancer. Cancer Discov. (2011) 1:78–89. 10.1158/2159-8274.CD-11-0005 22328973 PMC3274752

[44] Pirker R Pereira JR Szczesna A von Pawel J Krzakowski M Ramlau R. Cetuximab plus chemotherapy in patients with advanced non-small-cell lung cancer (FLEX): an open-label randomised phase III trial. Lancet. (2009) 373:1525–31. 10.1016/S0140-6736(09)60569-9 19410716

[45] Thatcher N Hirsch FR Luft AV SzHczesna A Ciuleanu TE Dediu M. Necitumumab plus gemcitabine and cisplatin versus gemcitabine and cisplatin alone as first-line therapy in patients with stage IV squamous non-small-cell lung cancer (SQUIRE): an open-label, randomised, controlled phase 3 trial. Lancet Oncol. (2015) 16:763–74. 10.1016/S1470-2045(15)00021-2 26045340

[46] Brahmer J Reckamp KL Baas P Crino L Eberhardt WE Poddubskaya E. Nivolumab versus Docetaxel in advanced squamous-cell non-small-cell lung cancer. N Engl J Med. (2015) 373:123–35. 10.1056/NEJMoa1504627 26028407 PMC4681400

[47] Herbst RS Baas P Kim DW Felip E Perez-Gracia JL Han JY. Pembrolizumab versus docetaxel for previously treated, PD-L1-positive, advanced non-small-cell lung cancer (KEYNOTE-010): a randomised controlled trial. Lancet. (2016) 387:1540–50. 10.1016/S0140-6736(15)01281-7 26712084

[48] Fehrenbacher L Spira A Ballinger M Kowanetz M Vansteenkiste J Mazieres J. Atezolizumab versus docetaxel for patients with previously treated non-small-cell lung cancer (POPLAR): a multicentre, open-label, phase 2 randomised controlled trial. Lancet. (2016) 387:1837–46. 10.1016/S0140-6736(16)00587-0 26970723

[49] Cappuzzo F McCleod M Hussein M Morabito A Rittmeyer A Conter HJ LBA53IMpower130: Progression-free survival (PFS) and safety analysis from a randomised phase III study of carboplatin + nab-paclitaxel (CnP) with or without atezolizumab (atezo) as first-line (1L) therapy in advanced non-squamous NSCLC. Ann Oncol. (2018) 29(Suppl. 8):mdy424.065 10.1093/annonc/mdy424.065

[50] Jotte RM Cappuzzo F Vynnychenko I Stroyakovskiy D Abreu DR Hussein MA et al. IMpower131: primary PFS and safety analysis of a randomized phase III study of atezolizumab + carboplatin + paclitaxel or nab-paclitaxel vs. carboplatin + nab-paclitaxel as 1L therapy in advanced squamous NSCLC. J Clin Oncol. (2018) 36(Suppl. 18):LBA9000 10.1200/JCO.2018.36.18_suppl.LBA9000

[51] Paz-Ares LG Luft A Tafreshi A Gumus M Mazieres J Hermes B Phase 3 study of carboplatin-paclitaxel/nab-paclitaxel (Chemo) with or without pembrolizumab (Pembro) for patients (Pts) with metastatic squamous (Sq) non-small cell lung cancer (NSCLC). J Clin Oncol. (2018) 36(Suppl. 15):105 10.1200/JCO.2018.36.15_suppl.105

[52] Socinski MA Jotte RM Cappuzzo F Orlandi F Stroyakovskiy D Nogami N. Atezolizumab for first-line treatment of metastatic nonsquamous NSCLC. N Engl J Med. (2018) 378:2288–301. 10.1056/NEJMoa1716948 29863955

[53] Gainor JF Shaw AT Sequist LV Fu X Azzoli CG Piotrowska Z. EGFR mutations and ALK rearrangements are associated with low response rates to PD-1 pathway blockade in non-small cell lung cancer: a retrospective analysis. Clin Cancer Res. (2016) 22:4585–93. 10.1158/1078-0432.CCR-15-3101 27225694 PMC5026567

[54] Lisberg A Cummings A Goldman JW Bornazyan K Reese N Wang T. A Phase II Study of pembrolizumab in EGFR-mutant, PD-L1+, tyrosine kinase inhibitor naive patients with advanced NSCLC. J Thorac Oncol. (2018) 13:1138–45. 10.1016/j.jtho.2018.03.035 29874546 PMC6063769

